# CD36-Mediated Fatty Acid Oxidation in Hematopoietic Stem Cells Is a Novel Mechanism of Emergency Hematopoiesis in Response to Infection

**DOI:** 10.20900/immunometab20220008

**Published:** 2022-03-14

**Authors:** Maria Maryanovich, Keisuke Ito

**Affiliations:** 1 Ruth L. and David S. Gottesman Institute for Stem Cell and Regenerative Medicine Research, Albert Einstein College of Medicine, Bronx, New York, NY 10461, USA; 2 Department of Cell Biology, Albert Einstein College of Medicine, Bronx, New York, NY 10461, USA; 3 Albert Einstein Cancer Center, Albert Einstein College of Medicine, Bronx, New York, NY 10461, USA; 4 Department of Medicine, Albert Einstein College of Medicine, Bronx, New York, NY 10461, USA; 5 Einstein Diabetes Research Center, Albert Einstein College of Medicine, Bronx, NY 10461, USA

**Keywords:** hematopoietic stem cells, infection, fatty-acid oxidation, CD36, oxidative phosphorylation, hematopoiesis

## Abstract

Hematopoietic homeostasis depends on the close regulation of hematopoietic stem cell (HSC) activity in the bone marrow. Quiescence and activation in response to stress, among other changes in state, are mediated by shifts in HSC metabolic activity. Although HSC steady-state metabolism is well established, the mechanisms driving HSC activation, proliferation, and differentiation in response to stress remain poorly understood. Here we discuss a study by Mistry et al. that describes a novel metabolic mechanism that fuels HSC activation and expansion. The authors show that to meet their metabolic needs in response to infection, hematopoietic stem and progenitor cells uptake free fatty acids from their microenvironment via CD36 to fuel fatty acid oxidation. These exciting findings suggest that in the context of infection, HSCs undergo a metabolic shift toward fatty acid metabolism that drives emergency hematopoiesis and raise questions about the role of the microenvironment in this process.

The life-long production of blood depends on the ability of hematopoietic stem cells (HSCs) to self-renew, differentiate, and form all hematopoietic lineages. HSCs rely upon precisely controlled interactions with their local microenvironment in the bone marrow (or “niche”) to preserve quiescence and maintain normal blood output [1]. These interactions are based upon membrane-bound, locally secreted, and/or long-range signals produced by a complex network of blood vessels, sympathetic nerve fibers, and hematopoietic and supporting stromal cells that control intrinsic properties of functional HSCs [1]. Inflammation is defined as a protective immune response, underlain by a variety of pathophysiological processes that are in part caused by infection and tissue injury/damage. In response to infection, quiescent HSCs are activated to proliferate and engage in blood formation, which replenishes immune effector cells [2]. Furthermore, pro-inflammatory cytokines in the inflamed microenvironment drive myeloid-biased differentiation and potentially contribute to the development of human clonal hematopoiesis [3].

Cellular metabolism has emerged as a critical regulator of HSC homeostasis, dysregulation of which can trigger changes in HSC cell cycle dynamics, leading to functional decline [4]. Quiescent HSCs maintain low metabolic activity and rely upon anaerobic glycolysis as their primary energy source in the hypoxic niche [5]; however, they maintain a relatively high mitochondria content and exhibit heightened mitochondrial activity [6–9]. In response to stress, HSCs leave the quiescent state and shift to aerobic metabolism, depending on mitochondrial oxidative phosphorylation (OXPHOS) to produce the ATP needed to meet the metabolic requirements associated with proliferation and differentiation (reviewed in [5]).

Fatty acid metabolism has emerged as a critical regulator of HSC self-renewal. Deletion of the fatty acid transport and oxidation regulator Peroxisome-proliferator activated receptor delta *(Ppard)* results in disrupted HSC asymmetric division, which leads to symmetric commitment of daughter cells, exhaustion, and poor repopulation capacity [10,11]. The identification of adipose tissue (AT) as a potential reservoir for functional HSCs [12] strengthens the notion that HSCs utilize fatty acid oxidation (FAO) to maintain homeostasis. In the context of hematopoietic malignancy, FAO is critical to supporting malignancy and maintenance of acute myeloid leukemia (AML) blasts [13]. Furthermore, FAO has been reported to play a crucial role in maintaining leukemic stem cells (LSCs) and supporting their metabolic needs to evade chemotherapy [14]. These studies suggest that, similar to LSCs, HSCs potentially rely on FAO for energy production in pathologic conditions [15]. As the bone marrow microenvironment tightly regulates stem cell activity, one could postulate that the interaction of HSCs with adipolineage cells affects their metabolic state. Numerous studies have reported that bone marrow adipocytes modulate HSC activity by secretion of factors such as adiponectin, dipeptidyl peptidase 4 (DPP4), and stem cell factor (SCF) [1,16–18]. However, it is unknown how these interactions affect HSC metabolism and whether they specifically affect lipid metabolism in HSCs. Thus, the intricate relationship between the extrinsic control of HSC activity by niche adipocytes and the intrinsic role played by lipid metabolism in HSC maintenance is complex (Figure 1). A better understanding of these mechanisms is urgently needed to improve stem-cell-based therapies targeting the bone marrow microenvironment.

In a recent study by Mistry et al. published in Nature Communications [19], the authors examine the role of FAO in HSC physiology and describe previously underappreciated, intrinsic roles for fatty acid metabolism in fueling hematopoietic response to infection. To model acute bacterial infection in vivo, the authors challenged mice with either Salmonella Typhimurium (*S. typhimurium*) or its outer membrane lipopolysaccharide (LPS), which led to rapid HSC and leukocyte expansion and elevation of free fatty acids (FFA) in the serum. Using a novel model to quantify fatty-acid uptake by hematopoietic cells, the authors revealed that challenging mice with either *S. typhimurium* or LPS triggered rapid uptake of FFA by hematopoietic stem and progenitor cells (HSPCs), and subsequent experiments confirmed that bacterial infection enhanced FAO in these activated HSPCs. Moreover, pharmacological inhibition of β-oxidation using etomoxir suppressed the expansion and proliferation of HSPCs in vivo. These findings establish that enhanced FAO induces metabolic remodeling that enables HSPCs to transition from steady-state to emergency hematopoiesis to combat infection. Fatty acid metabolism is thus a key mechanism supporting stem cell activation in response to infection-mediated physiological stress; however, it has yet to be determined whether this holds true for malignancies or stem cells in other tissues.

Schematic overview of the role of fatty acid metabolism and adipocytes in the maintenance of HSCs. Quiescent HSCs are kept in a low metabolic state and utilize glycolysis for energy production. HSC self-renewal and asymmetric division depend on FAO, mediated by PPARδ and PPARγ. To maintain homeostasis, bone marrow adipocytes negatively regulate HSC activity (orange box and arrows). Adipocyte-derived SCF has been shown to facilitate hematopoietic recovery following genotoxic stress, however, the role of FAO in hematopoietic regeneration is unknown (green box and arrows). Based on findings by Mistry et al. [19], bacterial infection leads to the accumulation of FFA that are taken up by HSCs and progenitor cells mediated by CD36 and IL6, leading to enhanced FAO and OXPHOS to accommodate hematopoietic expansion (red box and arrows). During aging, there is an increase in bone marrow adipogenesis that has been shown to have a negative impact on HSC function. Aging is associated with HSC expansion and old HSCs exhibit elevated OXPHOS. However, the role of FAO in HSC aging has yet to be determined (purple box). Figure created by Biorender.com.

To further understand the mechanisms that facilitate FAO in HSCs, the authors quantified the transcript and protein levels of known lipid transporter proteins and identified that the fatty-acid translocase CD36 was consistently upregulated in response to infection. As CD36 facilitates fatty-acid uptake and oxidation [20–22], these data imply that elevated CD36 levels contribute to the enhanced FAO detected in HSCs. Previous studies of blast crisis chronic myeloid leukemia (CML) have reported that elevated expression of CD36 in LSCs is associated with increased FAO and protection from chemotherapy [14,23]. In the context of bacterial infection, Mistry et al. demonstrate that either inhibition of CD36 with sulfosuccinimidyl oleate (SSO) or deletion of CD36 in mice (CD36^−^/^−^ mice) results in reduced FFA uptake, lipid content, basal and maximal respiration, and HSPC proliferation. Moreover, specific deletion of CD36 in hematopoietic cells revealed an intrinsic role for FAO in inducing the metabolic switch needed to sustain activation and subsequent proliferation of HSCs.

In summary, Mistry et al. report a new mechanism of emergency hematopoiesis and highlight FAO as a central metabolic pathway fueling HSPC expansion in response to infection. These findings shed light on the role of lipid metabolism in stem cell maintenance and raise new questions about the role of the microenvironment in this process. What is the source of FFA? The present study does not address how infection reshapes the bone marrow microenvironment to accommodate the availability of FFA, nor does it explore the bone marrow AT as a source for circulating FFA. Furthermore, exploring how induction of FAO in HSCs relates to other stress conditions and pathologies associated with enhanced adipogenesis would be of great interest. For example, in both aging and obesity, there is an increase in bone marrow adipogenesis, which is accompanied by poor HSC maintenance [16,24]. Aged HSCs exhibit enhanced OXPHOS levels [25], and future studies will be needed to determine the dependence of old HSCs on FAO. Furthermore, pro-inflammatory cytokine IL-6 was reported to trigger chronic myelomonocytic leukemia-like disease in Tet2 knockout models with age [26], and the current study by Mistry et al. showed that IL-6 contributes to FFA uptake in HSCs [19]. Still, it is tempting to postulate that aged HSCs hijack FAO to fuel their expansion and support age-related myeloid-biased differentiation. In contrast, a high-fat diet results in loss of functional HSCs and enhanced myelopoiesis [27,28]. This suggests that the high-fat content from such a diet elicits distinct effects in HSCs that lead to lineage skewing, which in turn potentiates inflammatory responses. Thus, the role of adipocytes and the effects of increased marrow fat content are specific to pathology as well as the type of endocrine signal (Figure 1). Further studies are needed to clarify how cues derived from adipolineage cells in the niche affect the metabolic activity of HSCs at both steady-state and in response to stress.

## Figures and Tables

**Figure 1.**
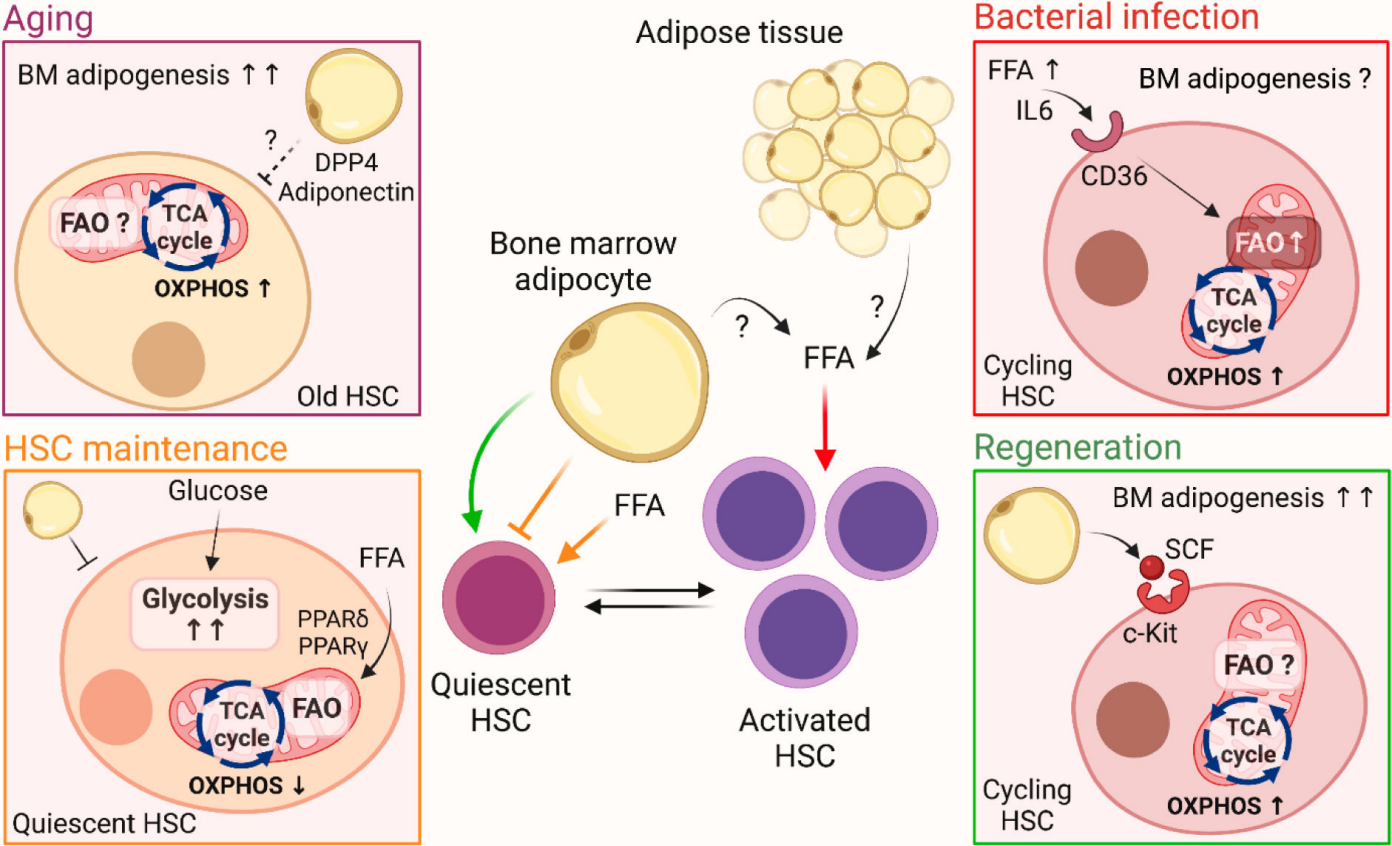
Overview of Fatty Acid Metabolism in HSCs.
